# Long-Term Supplementation of Royal Jelly (Raydel^®^) Improves Zebrafish Growth, Embryo Production and Survivability, Blood Lipid Profile and Functionality of Vital Organs: A 72-Weeks’ Consumption Study

**DOI:** 10.3390/ph17030324

**Published:** 2024-03-01

**Authors:** Kyung-Hyun Cho, Hyo-Seon Nam, Ashutosh Bahuguna, Ji-Eun Kim

**Affiliations:** Raydel Research Institute, Medical Innovation Complex, Daegu 41061, Republic of Korea

**Keywords:** royal jelly, antioxidant, high-density lipoprotein, liver, lipidemia, oxidative stress, ovary, testis, zebrafish

## Abstract

Royal jelly is a honeybee product with substantial pharmacological and health promotional activities. Nevertheless, the health implications associated with the prolonged dietary supplementation of royal jelly have yet to be elucidated extensively. Herein, 72 weeks of dietary supplementation of royal jelly at 5% and 10% (*w*/*w*) were investigated to assess the impact on zebrafish survivability, body weight, liver, testis, ovary functionality, and blood lipid profile. The results revealed no adverse effect of 72 weeks of royal jelly supplementation on zebrafish survivability. Conversely, a noteworthy enhancement in the zebrafish body weight was observed in royal-jelly-supplemented zebrafish in a concentration-dependent manner [5% and 10% (*w*/*w*)]. Interestingly, female zebrafish were found to be more biased, with a significant 17% (*p* < 0.001) and 23% (*p* < 0.001) higher body weight enhancement after 72 weeks of consumption of 5% and 10% (*w*/*w*) royal jelly, compared to the male zebrafish. The histological outcome revealed no sign of hepatotoxicity; moreover, diminished reactive oxygen species (ROS) and apoptosis were observed in the hepatic tissue of the royal-jelly-supplemented group. Consistent with the histological outcomes, the liver function biomarkers, aspartate aminotransferase (AST) and alanine aminotransferase (ALT), exhibited a significant decrease of 1.9-fold (*p* = 0.006) and 1.4-fold (*p* = 0.003) in zebrafish supplemented with royal jelly compared to those on a normal diet (ND) and zebrafish given supplements. Also, no sign of ovary and testis-related toxicity was observed in the royal-jelly-supplemented group during the 72-week period. Furthermore, the 10% (*w*/*w*) royal-jelly-consuming zebrafish exhibited a notable 2.1-fold increase (*p* = 0.018) in egg-laying ability compared to the ND-supplemented zebrafish. The 10% (*w*/*w*) royal jelly supplementation also effectively maintained the blood lipid profile by curtailing serum triglycerides (TG) and elevating high-density lipoprotein cholesterol (HDL-C). Conclusively, royal jelly dietary supplementation for a prolonged time found royal jelly to be safe to consume, to efficiently improve hepatic function, reproduction, and sexual health, and to augment the serum HDL-C level.

## 1. Introduction

Royal jelly is a milky white color, slightly pungent, mildly acidic (pH 3.6–4.2) viscous solution produced by the hypopharyngeal and mandibular glands of worker honeybees that are extensively consumed by the queen honeybee [1,2]. Typically, royal jelly is composed of water (50–60%), proteins (18%), carbohydrates (15%), lipids (3–6%), vitamins, trace elements, and free amino acids [3,4]. However, the composition may vary slightly according to the environmental conditions, harvesting season, floral source, etc., [5]. Proteins are the predominant constituent of royal jelly. The principal proteins present in royal jelly are known as major royal jelly proteins (MRJPs). The MRJPs are water-insoluble (49–87 kDa average molecular weight proteins) that are broadly categorized into nine distinct groups (MRJP1–MRJP9) [1,6]. Among the nine different MRJPs, MRJP1 is the principal protein, constituting 31–66% of total MRJPs [5,7]. In addition to diverse proteins, royal jelly is a rich natural source of essential free amino acids [4]. Lipids are another vital constituent of royal jelly, accounting for 3–6% of the total royal jelly composition. Among the distinct lipids present in the royal jelly, the majority of the lipids are composed of saturated and unsaturated fatty acids (8–12 carbon chains) with mono- or dicarboxylic acid groups that account for 80% of the total lipids in royal jelly. The most noteworthy fatty acid that is exclusively present in royal jelly is 10-hydroxy-2 decanoic acid (10-HDA), which is responsible for imparting several beneficial properties to royal jelly [4,8]. Besides fatty acids, the lipid content of royal jelly is constituted of wax (5–6%), phenolic acid (4–10%), and sterol (3–4%) [5]. The royal jelly also harbors a variety of trace metals, notably serving as an abundant reservoir of potassium (2462–3120 mg/kg) [9].

Royal jelly is well known to exert numerous pharmacological effects owing to its antioxidant, anti-inflammatory, and antimicrobial properties [5]. The noteworthy role of royal jelly as an antitumor, antiallergic, antiaging, wound-healing, and immunomodulatory agent is well documented in the literature [5,8,10,11]. Also, royal jelly is substantially used in the cosmetic industry due to its inhibitory effect on sebum secretion and, consequently, its impact on acne formation, acne-induced lesions, and small wounds [12]. Several preclinical and clinical studies demonstrated royal jelly’s therapeutic role against multiple sclerosis [13], traumatic brain injury [14], and infertility [15]. Nevertheless, the culminating effect of royal jelly was noted to alleviate premenstrual syndrome and menopause in females [16,17] and maintain the blood lipid profile [18].

Most of the studies conducted on royal jelly are confined to its therapeutic use, and limited sudies are available documenting a high amount of royal jelly consumpation for a long duration. Also, certain regulatory authorities, such as the European Food Safety Authority, have demarcated that existing evidence does not support the claim of health benefits obtained by consuming royal jelly [19].Therefore, in the present study, a high amount (5% and 10%) of royal jelly was supplemented with the normal diet and consumed for a long duration [72 weeks (~1.5 years)] by zebrafish to examine the impact on health, aiming to explore the true potential of royal jelly as a functional food.

Zebrafish was selected as a model organism owing to its shared genomic similarity with humans [20], which recognized zebrafish as a suitable organism for preclinical studies. Several reports have documented zebrafish as a canonical animal model for fertility research [21], dyslipidemia and associated disorders [22], toxicology, and drug discovery [23] due to its higher mechanistic resemblance to humans. Moreover, zebrafish have well-developed innate and acquired immune systems like vertebrates [24]. Also, zebrafish embryos are transparent, developed externally, and of a relatively large size, thus facilitating easy insertion of the compound of interest through microinjection and an evaluation of the developmental changes. Furthermore, the outcome of the many preclinical investigations conducted on zebrafish gathered substantial information that can help to design further human trials [25], underscoring the significance and suitability of zebrafish as a model organism.

The present study aims to evaluate the effect of a prolonged (over 72 weeks) dietary supplementation of royal jelly on zebrafish survivability, body weight, liver, ovaries, testis morphology, and blood lipid profile, in addition to the egg-laying behavior of the female zebrafish with a normolipidemic diet.

## 2. Results

### 2.1. In Vitro Antioxidant Activity of Royal Jelly

**Figure 1:**
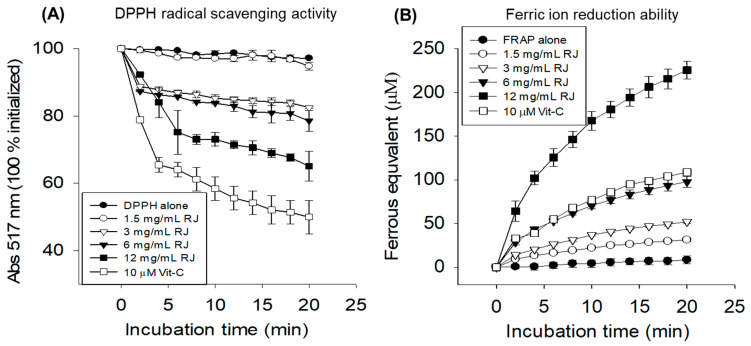
In vitro antioxidant activity of royal jelly. (**A**) DPPH free radical scavenging assay. (**B**) Ferric-ion-reducing antioxidant assay. Each value represents the mean ± SD of three independent experiments. RJ represents royal jelly, and Vit-C represents vitamin C (a positive control). RJ and Vit-C were dissolved in water. The indicated concentration represents the final amount of RJ and Vit-C treatment.

The antioxidant activity of royal jelly was assessed via DPPH free radical scavenging and FRAP assay. As depicted in Figure 1A, royal jelly efficiently scavenges DPPH free radicals in a time- and concentration-dependent manner. At 20 min incubation, 18.8%, 23.4%, and 36.2% DPPH free radical scavenging activity was observed at 3, 6, and 12 mg/mL royal jelly concentrations, respectively. The 18.2 mg/mL royal jelly concentration was found to be the inhibitory concentration fifty (IC_50_) for the DPPH free radicals.

Royal jelly also displayed a substantial ferric ion reduction ability in both a time- and concentration-dependent manner (Figure 1B). At 20 min incubation 31.4 ± 1.4 μM, 52.1 ± 2.1 μM, 97.8 ± 6.4 μM and 225.7 ± 10.0 μM ferrous ion equivalent was observed at 1, 3, 6 and 12 mg/mL of royal jelly concentrations. The outcome of the DPPH and FRA assay suggested a substantial antioxidant potential of royal jelly.

### 2.2. Protective Effect of Royal Jelly against H_2_O_2_ Exposure in Zebrafish Embryos

**Figure 2:**
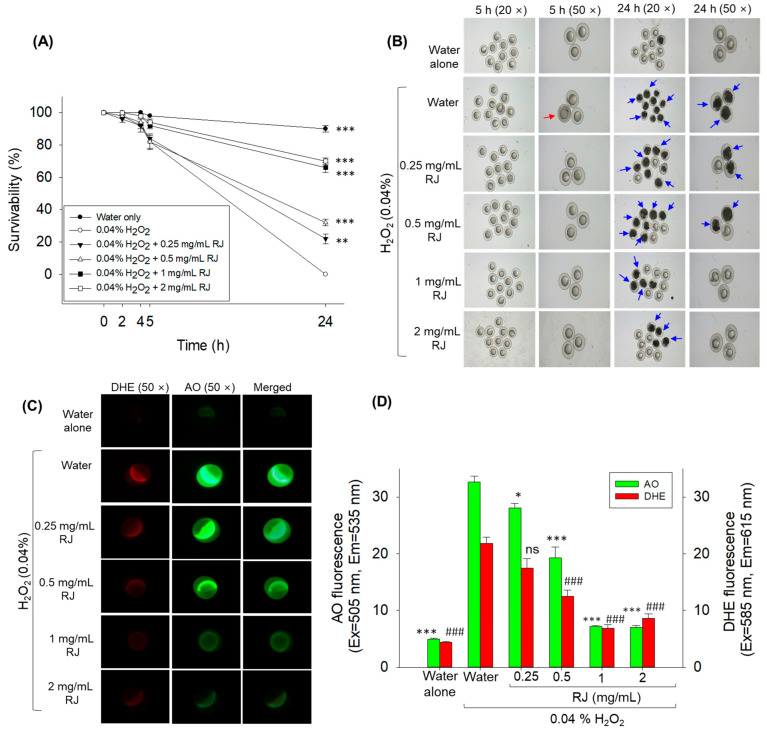
The effect of royal jelly on the hydrogen peroxide (H_2_O_2_)-induced stress in zebrafish embryos. (**A**) Kinetics of zebrafish embryo survivability 24 h post-treatment. (**B**) Images of zebrafish embryos at 5 and 24 h post-treatment (red arrow indicates swollen embryo as a sign of dying; blue arrow indicates dead embryos). (**C**) Fluorescent images of dihydroethidium (DHE)- and acridine orange (AO)-stained embryos (at 5 h post-treatment). (**D**) Image J-based quantification of DHE and AO fluorescent intensities. *, **, and *** represent the statistical difference at *p* < 0.05, *p* < 0.01, and *p* < 0.001 for AO fluorescent intensity, while ^###^ represents the statistical difference at *p* < 0.001 for DHE fluorescent intensity compared to the water + H_2_O_2_-treated group; ns represents the non-significant difference between the groups. RJ stands for royal jelly.

As depicted in Figure 2A,B, the embryo’s survivability in response to hydrogen peroxide (H_2_O_2_) exposure was severely affected in a time-dependent manner. At 5 h post-exposure to H_2_O_2_, the embryos’ survivability declined to 82%, and finally, the death of all embryos was observed at 24 h post-exposure to H_2_O_2_. On the contrary, royal jelly effectively mitigated H_2_O_2_-induced toxicity in a dose-dependent manner. The embryos treated with 0.25 and 0.5 mg/mL royal jelly showed 22% and 32% embryo survivability at 24 h post-treatment (Figure 2A,B). At the 1 and 2 mg/mL royal jelly concentration, 66% and 70% embryo survivability was observed, signifying the protective role of royal jelly against the external stress H_2_O_2_ causes in zebrafish embryos.

The outcome of the dihydroethidium (DHE) and acridine orange (AO) fluorescent staining revealed massive reactive oxygen species (ROS) production and a large extent of apoptosis in the embryos exposed to H_2_O_2_. The treatment of embryos with royal jelly effectively prevents the H_2_O_2_-derived ROS generation in a dose-dependent manner (Figure 2C,D). The zebrafish embryos treated with 0.5, 1, and 2 mg/mL royal jelly displayed a significantly reduced production of ROS, by 1.7-, 3.2-, and 2.6-fold (*p* < 0.001) lower than the ROS production observed in zebrafish embryos exposed to only H_2_O_2_. In line with ROS production, heightened apoptosis was observed in the embryos exposed to H_2_O_2_, which was significantly altered by the treatment with royal jelly in a dose-dependent manner (Figure 2C,D). A significantly lower apoptosis, by 1.2-fold (*p* < 0.05) and 1.7-fold (*p* < 0.001), was observed in the embryos treated with 0.25 and 0.5 mg/mL royal jelly compared to the H_2_O_2_-exposed embryos. Consistent with this, a 4.6-fold (*p* < 0.001) lower apoptosis was observed in 1 and 2 mg/mL royal-jelly-treated embryos than the only H_2_O_2_-exposed embryos.

### 2.3. Royal Jelly Prevents Carboxymethyllysine (CML)-Induced Acute Embryo Death

**Figure 3:**
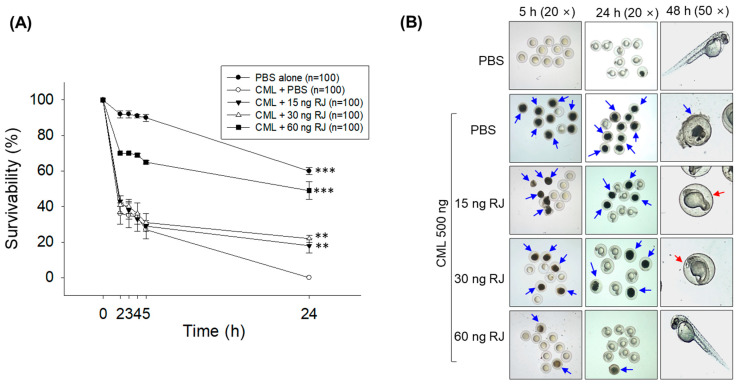

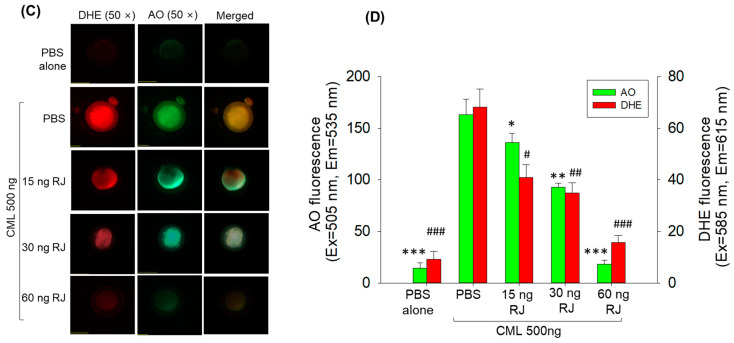
The effect of royal jelly on the survivability of zebrafish embryos when exposed to the acute toxicity posed by the microinjection of carboxymethyllysine (CML). (**A**) Survival kinetics of zebrafish embryos up to 24 h post-injection. (**B**) Images representing the zebrafish embryo survivability and developmental stage at 5, 24, and 48 h post-injection. The blue arrow indicates dead embryos, while the red arrow indicates unhatched embryos. (**C**) Fluorescent images of zebrafish embryos (at 5 h post-injection) stained with dihydroethidium (DHE) and acridine orange (AO). (**D**) Quantifying DHE and AO fluorescence levels in zebrafish embryos using Image J software 1.53r. Symbols *, ** and *** denote statistical significance at *p* < 0.05, *p* < 0.01, and *p* < 0.001, respectively, for AO fluorescent. Similarly, ^#^, ^##^ and ^###^ represent statistical significance at *p* < 0.05, *p* < 0.01, and *p* < 0.001 for DHE fluorescent intensity compared to the CML+PBS-treated group; ns indicates a non-significant difference between the groups. RJ refers to royal jelly.

The embryo survivability, as depicted in Figure 3A,B, was severely affected by the microinjection of carboxymethyllysine (CML, 500 ng). At 24 h post-injection, the highest embryo survivability (60%) was observed in the PBS-injected group. In contrast with this, embryo survivability declined periodically and reached zero (100% mortality) at 24 h post-injection in the CML-injected group (Figure 3A,B). The royal jelly treatment of precisely 60 ng displayed a substantial protective role in embryos against the toxicity caused by CML. The 60 ng royal-jelly-injected group exhibited a significantly higher survivability, at 49% (*p* < 0.001), than the only CML-injected group. However, the 60 ng, 15 and 30 ng royal jelly injections were found to be least effective in countering CML-posed adversity in embryos. In 15 and 30 ng royal-jelly-injected groups, embryo survivability declined periodically up to 5 h post-injection, which is nearly similar to the embryo survivability pattern observed in the CML-injected group. However, after 5 h post-injection, embryo survivability becomes stable in the 15 and 30 ng royal-jelly-injected group, which is substantially higher than the embryo survivability detected in the only CML-injected group. 

Most of the surviving embryos in the 60 ng royal-jelly-injected group displayed typical hatching without any sign of developmental deformities at 48 h post-injection (Figure 3B). Unlike this, normal embryo hatching was severely compromised in the 15 and 30 ng royal-jelly-injected groups. No embryo hatching was observed in the 15 and 30 ng royal-jelly-injected group at 48 h post-injection. The result signifies that the injection of royal jelly at a higher concentration (60 ng) effectively prevents CML-induced toxicity and developmental deformities in zebrafish embryos.

The results of DHE fluorescent staining observed at 5 h post-injection suggest a massive ROS production in the CML-injected group that is efficiently curtailed by the treatment of royal jelly in a dose-dependent manner (Figure 3C,D). Significantly lower ROS production, at 1.4 (*p* < 0.05), 1.9 (*p* < 0.01), and 4.3-fold (*p* < 0.05), was observed in 15, 30, and 60 ng royal-jelly-injected groups as compared to the CML+PBS-injected group. Consistent with the findings of DHE staining, the AO staining showed that royal jelly effectively prevents CML-induced apoptosis. The embryos that received 15, 30, and 60 ng royal jelly injection showed a 1.4 (*p* < 0.05), 1.9 (*p* < 0.01), and 4.3-fold (*p* < 0.05) lower AO fluorescent intensity than the CML-injected group, signifying the substantial anti-apoptotic role of royal jelly.

### 2.4. Body Weight and Survivability of Zebrafish

**Figure 4:**
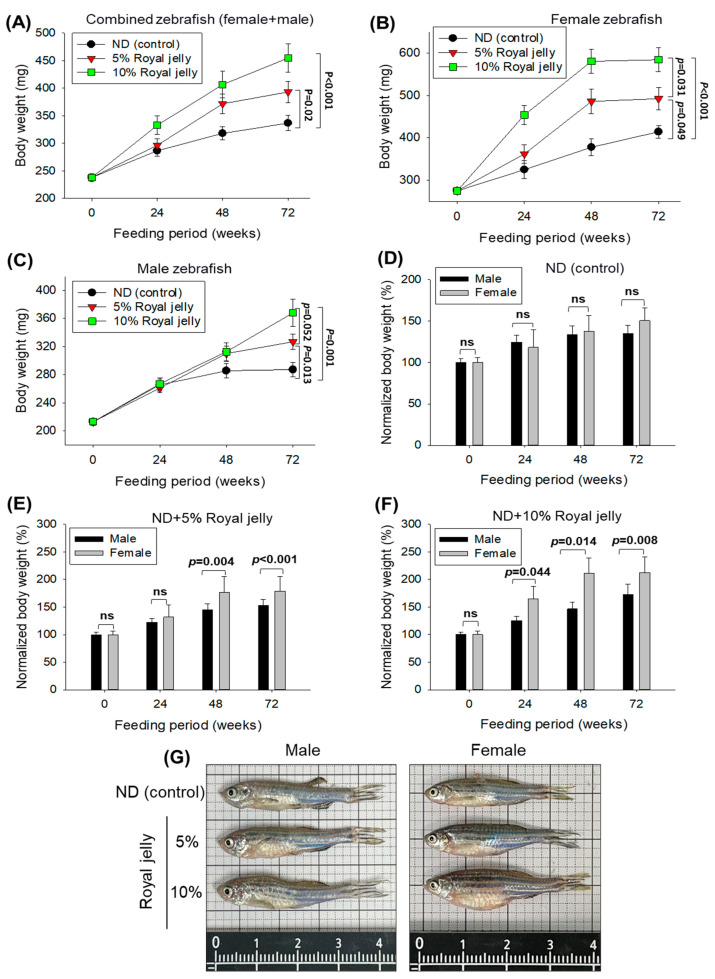
Long-term consumption of royal jelly on body weight of zebrafish. (**A**) A time-dependent (0–72 weeks) effect of 5% and 10% consumption of royal jelly on the body weight of zebrafish (female and male combined). (**B**,**C**) Time-dependent (0–72 weeks) effect of 5% and 10% consumption of royal jelly on the body weight of female and male zebrafish, respectively. (**D**–**F**) Comparative time-dependent body weight changes between female and male in control, and 5% and 10% royal-jelly-supplemented groups, respectively. The normalized body weight at different time points was calculated with respect to 0-day body weight (considering this as 100%). (**G**) Representative images of male and female zebrafish from control, 5%, and 10% royal jelly consumption for 72 weeks. ND zebrafish received normal diet (ND), while zebrafish in 5% and 10% royal jelly groups received ND supplemented with 5% and 10% royal jelly. The *p*-value denotes the one-way ANOVA following Tukey’s Post Hoc analysis based on statistical differences between the groups; ns indicates non-significant differences between the groups.

As depicted in Figure 4A, the body weight increased with time in all the groups, though the most noteworthy changes were observed in the 10% royal-jelly-supplemented groups. At 24 weeks of feeding, 332.8 ± 16.9 mg bodyweight was observed in the 10% royal-jelly-supplemented group, which is 39.2% higher than the body weight observed at the beginning (238.0 ± 6.7 mg). Contrary to this, in 5% royal-jelly- and ND-supplemented groups, a ~20% body weight enhancement was observed compared to the initial body weight. At 48 weeks, the 10% royal-jelly-supplemented groups achieved the maximum weight (406.6 ± 24.1 mg), i.e., 9.3% and 27.7% higher than the body weight observed in 5% royal-jelly- and ND-supplemented groups. At the final 72 weeks, the highest body weight was observed in the 10% royal-jelly-supplemented group, followed by the 5% royal-jelly-supplemented group, 35.1% (*p* < 0.001) and 16.7% (*p* = 0.02) higher than the body weight observed in the ND-supplemented group.

The effect of royal jelly consumption on female and male zebrafish body weight was individually determined over 72 weeks (Figure 4B,C). An enhancement in body weight was observed among all the groups; however, the royal-jelly-supplemented female zebrafish displayed a vigorous body weight enhancement in a dose- and time-dependent manner (Figure 4B). At 72 weeks, a 1.5-fold, 1.8-fold, and 2.1-fold higher body weight was observed in ND, 5%, and 10% royal-jelly-supplemented female zebrafish, respectively, compared to the initial weight. However, over the 72 weeks, the maximum body weight was noticed in female zebrafish supplemented with 10% royal jelly, i.e., weight was 1.2-fold (*p* = 0.031) and1.4-fold (*p* < 0.001) higher than the body weight of 5% royal-jelly- and ND-supplemented groups (Figure 4B).

In contrast to the female zebrafish, the body weight of male zebrafish across the three groups remained almost similar up to 24 weeks (Figure 4C). Further, a slight increase in body weight was observed in both 5% and 10% royal-jelly-supplemented groups compared to the ND-supplemented group at 48 weeks. However, a significant enhancement of 1.3-fold (*p* = 0.001) and 1.2-fold (*p* = 0.013) in body weight in the 10% royal-jelly- and 5% royal-jelly-supplemented groups was observed compared to the ND-supplemented group at 72 weeks.

Furthermore, a comparative study between female and male body weight changes during the supplementation of ND, 5%, and 10% royal jelly revealed a non-significant effect of ND supplementation on the body weight of males and females during the 72 weeks (Figure 4D). In contrast to this, the 5% royal-jelly-supplemented group showed a 21.6% (*p* = 0.004) and 16.9% (*p* < 0.001) higher body weight in females compared to the males at 48 weeks and 72 weeks of consumption (Figure 4E). The most noteworthy effect on the female body weight enhancement was observed in the 10% royal-jelly-supplemented group (Figure 4F). At 24, 48 and 72 weeks supplementation of 10% royal jelly, the female body weight enhanced significantly, by 31.8% (*p* = 0.004), 44.1% (*p* = 0.014), and 23.2% (*p* = 0.008), compared to the male body weight at the respective time points. The results signify that royal jelly has a substantial effect on body weight enhancement, and female zebrafish are more vulnerable to this than male zebrafish.

Importantly, no adverse effect of royal jelly at the tested concentrations of 5% and 10% was observed on the survivability of zebrafish. A total of 77.5% survivability was observed in 5% and 10% royal-jelly-supplemented groups at 72 weeks, which was similar to the survivability observed in the ND-supplemented group, attesting to the safe nature of royal jelly consumption for the prolonged time.

### 2.5. Evaluation of the Liver

**Figure 5:**
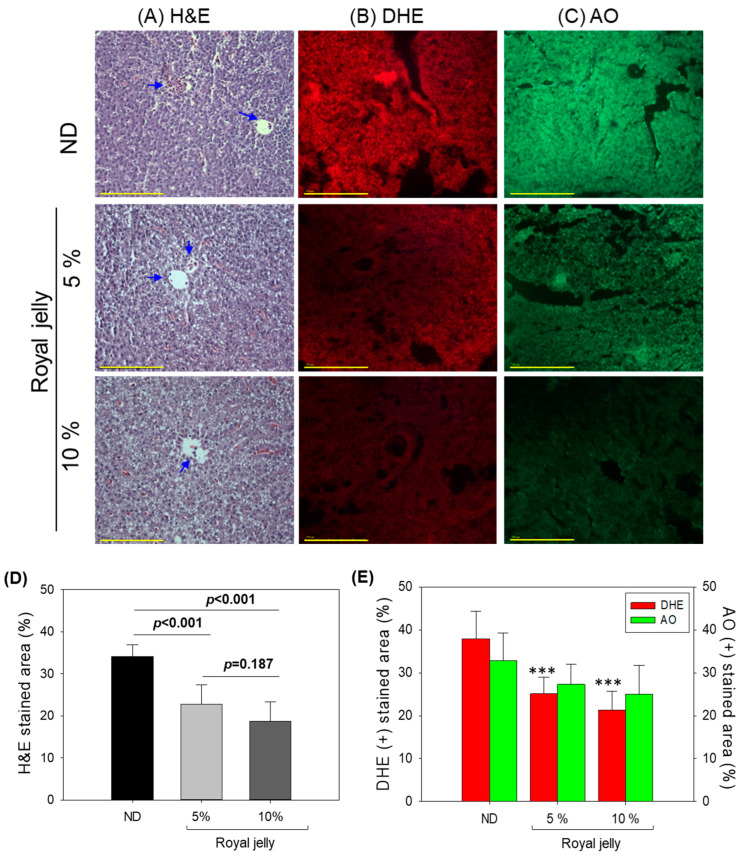
Effect of 72 weeks’ consumption of royal jelly on hepatic tissue of zebrafish. (**A**) Hematoxylin and eosin (H&E) staining images. Blue arrow indicate the infiltration of neutrophils. (**B**) Dihydroethidium (DHE) and (**C**) acridine orange (AO) fluorescent staining, respectively. [Scale bar, 100 μm]. (**D**,**E**) DHE- and AO-stained area quantification using Image J software (http://rsb.info.nih.gov/ij/, assessed on 30 January 2023). ND zebrafish received normal diet (ND), while zebrafish in 5% and 10% royal jelly groups received ND supplemented with 5% and 10% royal jelly. The *p*-value denotes the one-way ANOVA following Tukey’s post hoc analysis based on statistical differences between the groups. *** represents the statistical difference at *p* < 0.001 compared to the ND-supplemented group.

The H&E staining of the hepatic tissue extracted from ND- and royal-jelly-supplemented groups showed no sign of hepatic degeneration (Figure 5A). In all the groups, no visible signs of hepatic ballooning, lipid accumulation, and hepatic fibrosis were observed. Also, the results demonstrated no sign of excessive neutrophil infiltration around the portal vein across all the groups; however, as compared to the 5% and 10% royal jelly supplemented groups, a slightly higher neutrophil around the portal vein was observed in the ND-supplemented group. As depicted in Figure 5D, 22% and 18% H&E-stained areas were quantified in the 5% and 10% royal-jelly-supplemented groups which are 1.6-fold (*p* < 0.001) and 1.9-fold (*p* < 0.001) lower than the H&E-stained area observed in the ND-supplemented group, signifying the influence of royal jelly on hepatic health.

The ROS level in the hepatic tissue of the zebrafish after 72 weeks’ consumption of royal jelly was evaluated by DHE fluorescent staining (Figure 5B,E). The results revealed a significantly higher ROS level in the ND-supplemented group, i.e., 1.5-fold (*p* < 0.001) and 1.8-fold (*p* < 0.001) higher than the DHE-stained area in the 5% and 10% royal-jelly-supplemented groups. Comparing the 5% and 10% royal-jelly-supplemented groups, a non-significant effect on the ROS level was observed.

The AO fluorescent staining did not indicate any harmful impact of royal jelly (at the tested concentrations of 5% and 10%) on the extent of hepatic apoptosis when compared to the ND-supplemented group (Figure 5C,E). Nevertheless, a marginal decrease in the AO-stained area was noted in the 5% and 10% royal-jelly-supplemented groups in comparison to the AO-stained area measured in the ND-supplemented group, although this difference between the groups was not statistically significant (*p* > 0.05).

The histology results attest no adverse effect of prolonged royal jelly consumption on the liver; furthermore, royal jelly was found to be effective in minimizing the hepatic ROS level.

### 2.6. Hepatic Function Biomarkers

**Figure 6:**
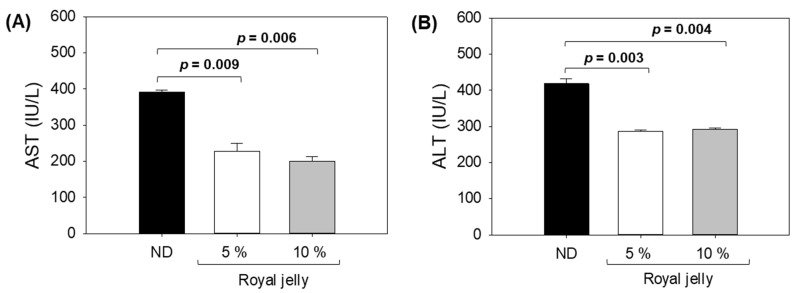
Effect of 72 weeks consumption of royal jelly on (**A**) aspartate aminotransferase (AST) and (**B**) alanine aminotransferase (ALT) hepatic function biomarkers. ND zebrafish received normal diet (ND), while zebrafish in 5% and 10% royal jelly groups received ND supplemented with 5% and 10% royal jelly. The *p*-value denotes the one-way ANOVA following Tukey’s Post Hoc analysis based on statistical differences between the groups.

The hepatic function was assessed after 72 weeks of the consumption of ND and royal jelly by measuring serum AST and ALT levels. Figure 6A illustrates that the ND-supplemented group exhibited the highest AST levels, 1.7-fold (*p* = 0.009) and 1.9-fold (*p* = 0.006) higher than the AST levels quantified in the 5% and 10% royal-jelly-supplemented groups. In comparison to the 5% royal-jelly-supplemented group, the 10% royal-jelly-supplemented group exhibited a 12.4% lower AST level, though the difference was statistically non-significant (*p* > 0.05).

Consistent with the AST level, the maximum ALT level was detected in the ND-supplemented group, which is significantly (1.4-fold (*p* = 0.003)) higher than the ALT level detected in the 5% and 10% royal-jelly-supplemented groups.

The outcome of the hepatic function biomarkers is aligned with the hepatic histology results attesting to the non-hepatotoxic nature of royal jelly. The results strongly advocate a positive effect of royal jelly on the functionality of the liver.

### 2.7. Effect of Royal Jelly Consumption on Ovaries

**Figure 7:**
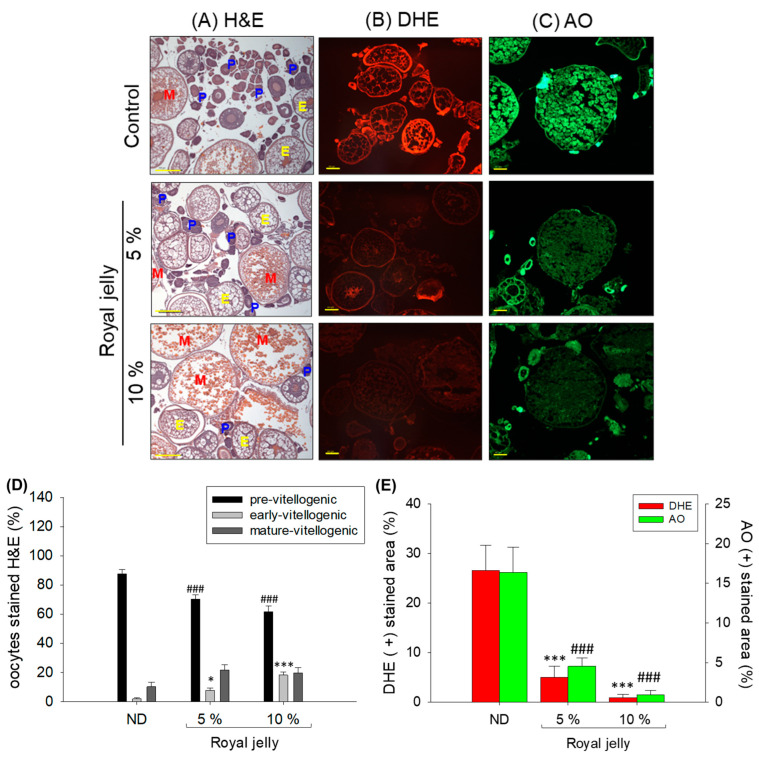
Effect of 72 weeks’ consumption of royal jelly on the ovary of zebrafish. (**A**) Hematoxylin and eosin (H&E)-stained images (P, E, and M indicating pre-vitellogenic, early-vitellogenic, and mature-vitellogenic stages, respectively). (**B**,**C**) dihydroethidium (DHE) and acridine orange (AO) fluorescent staining, respectively. [Scale bar, 100 μm]. (**D**) Quantification of different developmental stages of oocytes. (**E**) Quantification of DHE- and AO-stained area using Image J software. ND zebrafish received normal diet (ND), while zebrafish in 5% and 10% royal jelly groups received ND supplemented with 5% and 10% royal jelly. The *p*-value denotes the one-way ANOVA following Tukey’s Post Hoc analysis based on statistical differences between the groups. ^###^ represent *p* < 0.001 for pre-vitellogenic compared to ND, while * represent *p* < 0.05 and *** represent *p* < 0.001 for early-vitellogenic compared to ND. *** and ^###^ in (**E**) represents *p* < 0.001 compared to ND for DHE- and AO-stained areas, respectively.

The ovary’s histology, as depicted in Figure 7, showed the different oocyte populations in the ND, 5%, and 10% royal-jelly-supplemented groups. In the ND-supplemented group, a higher prevalence of previtellogenic oocyte was determined, significantly (1.2-fold and 1.2-fold_ higher than the previtellogenic oocyte observed in the 5% and 10% royal-jelly-supplemented groups. Contrary to this, in the royal-jelly-supplemented group, a higher population of early and mature vitellogenic oocytes were noticed. As compared to the ND-supplemented group, 4-fold and 11-fold higher early vitellogenic oocytes were observed in the 5% and 10% royal-jelly-supplemented groups. Likewise, ~1.9-fold higher mature vitellogenic oocytes were observed in the 5% and 10% royal-jelly-supplemented groups as compared to the ND-supplemented group.

The DHE staining of the ovary section revealed a diminished ROS level in the royal-jelly-supplemented group compared to the ND-supplemented group (Figure 7B,D). A significantly, 5-fold and 25-fold, lower ROS production was detected in the 5% and 10% royal-jelly-supplemented groups than the ND-supplemented group. The ovary ROS levels were affected by royal jelly supplementation in a dose-dependent manner, as is evident in the 5-fold lower ROS level in the 10% royal-jelly-supplemented group than the ROS levels observed in the 5% royal-jelly-supplemented group.

Consistent with the DHE staining, a lower extant of apoptosis as examined by AO staining was observed in the royal-jelly-supplemented groups than the ND-supplemented group (Figure 7C,D). A significant 3.6-fold and 16-fold lower apoptosis was detected in 5% and 10% royal-jelly-supplemented groups compared to the ND. As compared to the 10% royal-jelly-supplemented group, a 4.5-fold higher apoptosis was observed in 5% royal-jelly-supplemented group, suggesting the dose-dependent effect of royal jelly on diminishing apoptosis.

Combined histology and fluorescent staining outcomes showed a markable effect of royal jelly consumption on the morphology, as well as diminishing ROS levels and apoptosis.

### 2.8. Effect of Royal Jelly Consumption on Testis

**Figure 8:**
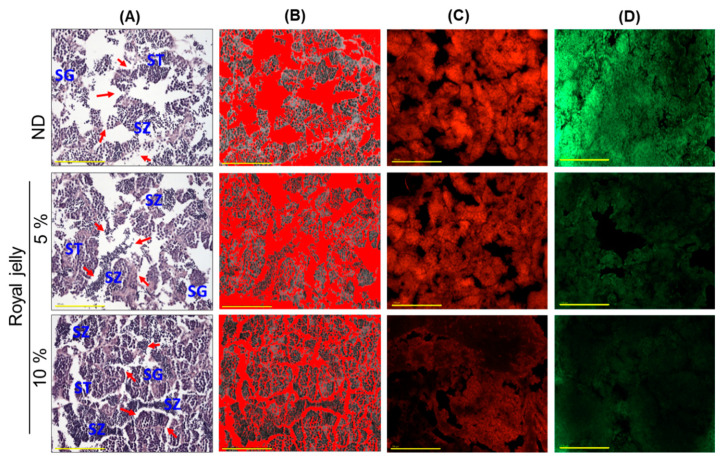

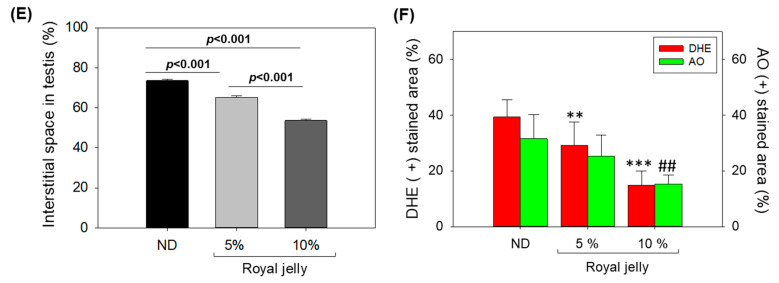
Effect of 72 weeks’ consumption of royal jelly on testis of zebrafish. (**A**) Hematoxylin and eosin (H&E)-stained images (ST, SG and SZ denote spermatocytes, spermatogonia, and spermatozoa). The red arrow indicates the void space between seminiferous tubules. (**B**) White area (void space) appeared in H&E staining, interchanged with red color at the 220–225 threshold value for the white color using Image J software). (**C**,**D**) dihydroethidium (DHE) and acridine orange (AO) fluorescent staining, respectively. [Scale bar, 100 μm]. (**E**,**F**) quantification of DHE- and AO-stained areas using Image J software. ND zebrafish received normal diet (ND), while zebrafish in 5% and 10% royal jelly groups received ND supplemented with 5% and 10% royal jelly. The *p*-value denotes the one-way ANOVA following Tukey’s Post Hoc analysis based on statistical differences between the groups. ** represent *p* < 0.01 and *** represent *p* < 0.001 compared to ND for DHE-stained area, while ^##^ represent *p* < 0.01 compared to ND for AO-stained area.

The H&E staining, as depicted in Figure 8A, showed the testicular histology of zebrafish supplemented with ND and royal jelly. The ND-supplemented group showed a loosely arranged tubular structure with sparsely populated spermatocytes and spermatogonia. Also, a broad gap between seminiferous tubules was observed across the testis section (Figure 8A,B,E). In contrast to this, in the royal jelly, mainly in 10% supplemented groups, a tightly arranged tubular structure with densely packed spermatocytes and spermatozoa was detected. Compared to the ND-supplemented group, in the 5% and 10% royal-jelly-supplemented groups, 1.2-fold (*p* < 0.001) and 1.4-fold (*p* < 0.001) lower interstitial space was observed between seminiferous tubules. Compared to the 5% group, the 10% royal-jelly-supplemented group showed a well-defined and densely packed spermatocyte and spermatozoa, along with a 1.2-fold (*p* < 0.001) reduced interstitial space between the seminiferous tubules, signifying the dose dependent effect of royal jelly on the testis.

The DHE staining showed 1.4-fold and 2.6-fold reduced ROS levels in the testicular section of the 5% and 10% royal-jelly-supplemented groups compared to the ND-supplemented group (Figure 8C,F). Compared to the 5% group, the 10% royal-jelly-supplemented group showed an effective 1.9-fold reduction in the ROS level, testifying to the dose-dependent effect of royal jelly on alleviating the ROS level. Similar to the DHE staining, the AO staining suggested a 2.1-fold reduction in apoptosis in the 10% royal-jelly-supplemented groups (Figure 8D,F). The results of the testicular histology support the beneficial effect of royal jelly consumption on testis morphology and the elimination/prevention of ROS generation and apoptosis.

### 2.9. Effect of Royal Jelly Consumption on Egg-Laying Ability

**Figure 9:**
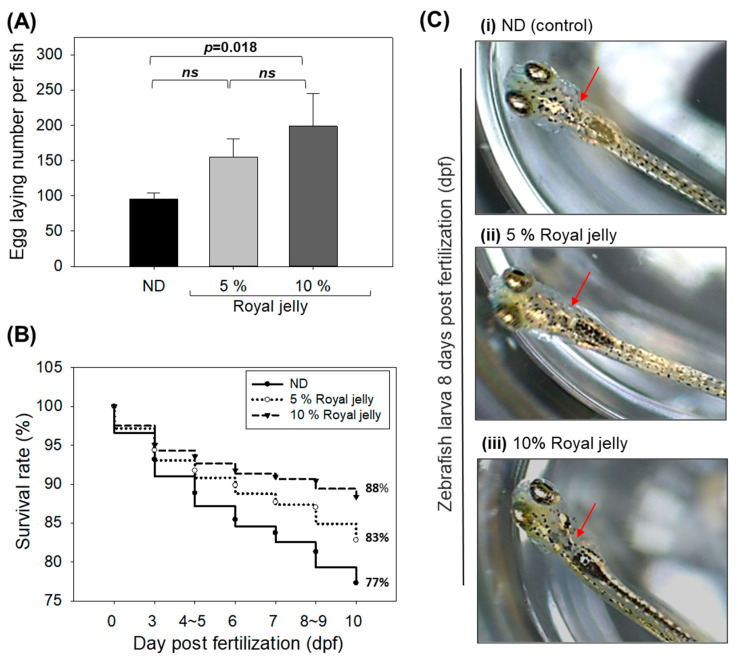
Effect of 72 weeks’ royal jelly consumption on the egg-laying activity of zebrafish. (**A**) Quantification of the laid eggs. ns, non-significant. (**B**) Survivability of the embryos 10 days post-fertilization (dpf). (**C**) Images of zebrafish larva at 8 days post-fertilization (dpf). The red arrow indicates the pigment formation. ND zebrafish received normal diet (ND), while zebrafish in 5% and 10% royal jelly groups received ND supplemented with 5% and 10% royal jelly. The *p*-value denotes the one-way ANOVA following Tukey’s Post Hoc analysis based on statistical differences between the groups.

As depicted in Figure 9A, royal jelly supplementation at 5% and 10% has a modulatory effect on the egg-laying behavior of zebrafish. The egg-laying ability of the 5% royal-jelly-supplemented group was enhanced by 1.6-fold compared to the ND-supplemented groups. However, the 10% royal-jelly-supplemented group showed the most noteworthy effect on the egg-laying activity, evident by the 2.1-fold (*p* = 0.018) increase in the number of laid eggs compared to the ND-supplemented group. Compared to the 5% group, the 10% royal-jelly-supplemented group showed a 1.3-fold increase in the number of laid eggs. Furthermore, royal jelly also displayed a substantial effect on the survivability of the fertilized eggs (embryos). As depicted in Figure 9B, 83% and 88% embryo survivability was observed in the 5% and 10% royal-jelly-supplemented groups compared to the 77% survivability observed in the ND-supplemented group. Among all the groups, no significant changes were observed in embryonic developmental deformities; however, the majority of embryos obtained for the 5% and 10% royal-jelly-supplemented groups displayed a deep black pigmentation on the head and the neck area (Figure 9C). The results demonstrate that royal jelly consumption has no adverse effect on the egg-laying activity of zebrafish; furthermore, royal jelly can positively modulate the egg-laying behavior and survivability of zebrafish embryos.

### 2.10. Blood Lipid Profile

**Figure 10:**
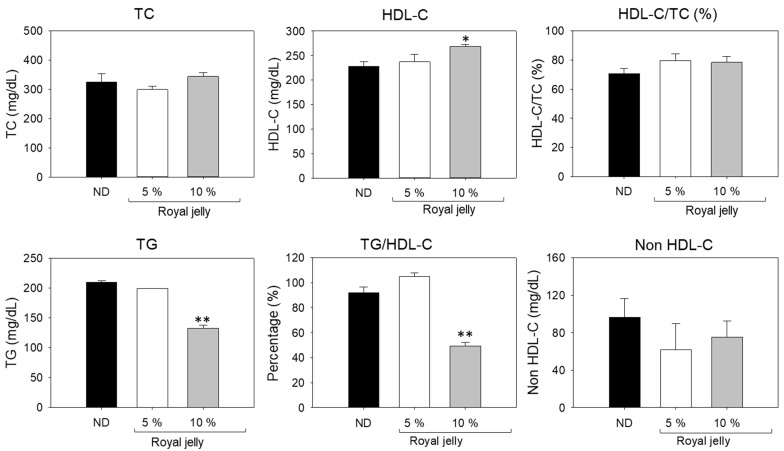
Impact of royal jelly consumption for 72 weeks on blood lipid profile of zebrafish. Total cholesterol (TC), triglycerides (TG), and high-density lipoprotein cholesterol (HDL-C). ND zebrafish received normal diet (ND), while zebrafish in 5% and 10% royal jelly groups received ND supplemented with 5% and 10% royal jelly. The *p*-value denotes the one-way ANOVA following Tukey’s Post Hoc analysis based on statistical differences between the groups. * and ** represents *p* < 0.05 and *p* < 0.01 for early vitellogenic compared to ND.

As shown in Figure 10, no significant change in TC levels was observed between the ND group and the group supplemented with royal jelly. In contrast to this, the HDL-C level was significantly changed between the groups. The maximum HDL-C level was detected in the 10% royal-jelly-supplemented group, which is 1.2-fold higher than the HDL-C level quantified in the 5 ND-supplemented group. The TG level significantly varied between the ND-, 5% royal-jelly- and 10% royal-jelly-supplemented groups. The lowest TG level was detected in the 10% royal-jelly-supplemented group, which was 36.8% and 32.2% lower than the TG levels quantified in the ND- and 5% royal-jelly-supplemented groups, respectively. Consistent with TG, the TG/HDL-C level was found to be the lowest in the 10% royal-jelly-supplemented group, which is 46.1% and 52.8% lower than the TG/HDL-C levels detected in the ND- and 5% royal-jelly-supplemented groups. No significant change in non-HDL-C levels was observed in ND-, 5%, and 10% royal-jelly-supplemented groups; however, the minimum amount was quantified in the 10% royal-jelly-supplemented group. The combined results reveal that the extended dietary supplementation of royal jelly does not exert any negative impact on the blood lipid profile; in fact, royal jelly was found to be effective in fostering the blood lipid profile of zebrafish, in spite of the body weight increase.

## 3. Discussion

Royal jelly is a rich source of various nutrients and pharmacologically important substances and is often used as a traditional medicine [7]. Several previous reports documented royal jelly’s therapeutic effect and functionality as an antioxidant, anti-inflammatory, antiaging, antimicrobial, and wound-healing agent [5,8,10,11], making it a viable commercial product. Despite its numerous uses, the health impact of the prolonged dietary intake of royal jelly needs to be extensively documented. In view of the above, royal jelly dietary supplementation in zebrafish for 72 weeks was tested to determine the health implications.

Prior to evaluating the dietary supplementation, the in vitro antioxidant activity and the protective effect of royal jelly on zebrafish embryos were determined upon exposure to external stress induced by exposure to H_2_O_2_ and the injection of CML. CML, classified as an advanced glycation end product (AGE), is widely recognized for its toxicity, resulting from the initiation of ROS/oxidative stress [26,27], ultimately leading to apoptosis. The outcomes suggested a substantial DPPH free radical scavenging and ferric ion reduction activity, signifying the antioxidant activity of royal jelly. The results align with a previous study deciphering the DPPH free radical scavenging activity of royal jelly [28,29,30,31]. In another recent study, the free radical scavenging activity of major royal jelly proteins (MRJPs) was also detected, substantially protecting against DNA damage from oxidative stress [32]. In the present work, we also observed the protective role royal jelly can play n zebrafish embryos against H_2_O_2_- and CML-induced oxidative stress. The protective effect of zebrafish embryos is strengthened by the antioxidant properties of royal jelly, which can effectively counter the ROS-induced oxidative stress and prevent apoptotic cell death in zebrafish embryos. The findings agree with a previous report suggesting the effective role of royal jelly in improving the cellular antioxidant, consequently enhancing the survivability of *Drosophila melanogaster* [33]. Similar to this, royal jelly displayed a protective role against sodium-fluoride-induced oxidative stress in mice by upregulating the cellular antioxidants superoxide dismutase (SOD) and catalase [34]. These earlier reports [32,33,34] strengthen the current findings and establish the important antioxidant role of royal jelly, which not only directly scavenges the free radicals but also has a substantial effect on the cellular antioxidant, and thus has a preventive effect against oxidative-stress-induced detrimental effects.

Further, the outcome of the long-term royal jelly consumption study revealed no adverse effect of royal jelly on the survivability of zebrafish, underscoring the royal jelly’s safety for consumption. Interestingly a substantial effect of royal jelly consumption on body weight enhancement was observed. The findings also revealed that females are more inclined towards weight gain compared to male zebrafish. The exact reason behind the body weight gain and its more notable effects in female zebrafish is not known. The most probable explanation for the body weight enhancement may be royal jelly’s effect on improving zebrafish’s metabolic process, similar to that in a bee colony to stimulate and increase larva growth [35]. However, further study is required to determine the exact reason behind the body weight enhancement achieved by the consumption of royal jelly and its bias toward female zebrafish. 

The consumption of royal jelly was not shown to lead to signs of hepatotoxicity. Moreover, the outcome regarding hepatic morphology and hepatic function biomarkers (AST and ALT) revealed a beneficial effect of royal jelly consumption on the hepatic health of zebrafish. The most probable reason behind the decrease in the AST and ALT levels in the royal-jelly-fed group compared to the ND-fed group is the variation in the TG levels in these groups (Figure 9). The consumption of royal jelly is associated with decreased TG levels, which could potentially influence the reduction in AST and ALT levels. An earlier published report [36] indicated a correlation between higher TG levels and elevated AST and ALT levels, further supporting this notion. However, further study is required to reach any conclusions. In addition to hepatic morphology and hepatic function biomarkers, royal jelly consumption substantially inhibited ROS generation and apoptosis in the hepatic tissue. The major reason behind the better hepatic health that was seen in response to royal jelly consumption lies in its antioxidant nature, which minimizes the cellular ROS and consequently impacts oxidative-stress-induced apoptosis and the functionality of the liver. This notion is strongly supported by previous studies documenting the antioxidant nature of royal jelly as a key event in preventing hepatic damage against varied forms of external stress [37]. The regulatory impact of royal jelly on the expression of liver antioxidants such as glutathione S-transferase (GST) and glutathione peroxidase (GSH-px) has been documented [37,38], which helps in hepatoprotection against oxidative-stress-induced damage. Royal jelly’s impact on upregulating nuclear factor erythroid 2-related factor 2 (Nrf2) has been documented, and royal jelly can consequently impact the expression of the important antioxidant enzymes that prevent hepatic damage [37,39].

We also observed low hepatic apoptosis in royal-jelly-supplemented zebrafish, attributed to the antioxidant properties of royal jelly, which can scavenge harmful ROS, which are key contributors to cellular apoptosis [40] Furthermore, the influence of royal jelly on the upregulation of the antiapoptotic Bcl-2 and downregulation of proapoptotic Bax and caspase 3 has been documented to inhibit apoptosis in the liver [39]. These findings also support the present findings regarding royal-jelly-supplemented zebrafish under similar conditions, where supplementation was found to inhibit apoptosis, and improve hepatic health.

Like the liver, no toxicity from royal jelly consumption (for 72 weeks) was noticed regarding the morphology and functionality of the ovaries and testes. Furthermore, it was observed that royal jelly enhances the functionality of the testes and ovaries, exerting a distinct influence on egg-laying behavior. The histological results suggest a diminished ROS and apoptosis in the testes and ovaries of the royal-jelly-supplemented zebrafish due to the antioxidant nature of royal jelly, improves the functionality of the testes and ovaries. This aligns well with previous findings suggesting a beneficial role of royal jelly in reproductive health [10] due to its ability to counter oxidative stress [41] Also, it has been reported that royal jelly impacts oxidative stress and modulates Bax and Bcl-2, leading to the protection of oocytes. Additionally, the impact of royal jelly (mainly imparted by 10-HDA) on reducing the follicle-stimulating hormone (FSH) and luteinizing hormone (LH) is another important reason for the improvements in ovarian health [10].

Royal jelly consumption was found to enhance body weight, which is often associated with dyslipidemia. In the present study, we found no adverse effect of prolonged royal jelly consumption on the disturbed blood lipid profile despite the significant impact on body weight. Moreover, royal jelly supplementation enhanced the HDL-C levels and reduced the TG levels. As oxidative stress and inflammation are the major culprits that disturb the lipid profile and cause dyslipidemia [42], we believe that the enhancement of the HDL-C levels and reductions in the TG levels might be linked with the antioxidant and anti-inflammation activity of royal jelly. A previously conducted study also suggested an impact of royal jelly consumption (150 mg/day) for 3 months (~12 weeks) on reducing TC, TG and LDL-C, and the elevation of HDL-C levels [18], strengthening the present findings.

## 4. Materials and Methods

### 4.1. Materials

Royal jelly was processed and analyzed by Raydel^®^ (Raydel Australia Pty, Ltd., Thornleigh, Sydney, NSW, Australia). A detailed specification of the royal jelly is provided in Supplementary Table S1. To prepare a royal-jelly-enriched diet, normal tetrabit (Tetrabit Gmhb D49304, Melle, Germany), a regular zebrafish diet, was mixed individually with 5% and 10% (*w*/w) royal jelly. The royal-jelly-supplemented normal tetrabit was blended vigorously to ensure the proper distribution of royal jelly among normal tetrabit.

Methylene blue (Cat#M4195), diphenyl-1-picrylhydrazyl (Cat#9286), 2 phenoxyethanol (Cat#P1126), acridine orange (Cat#A9231), dihydroethidium (Cat#A37291), and *N-ε-*carboxymethyllysine (Cat#114580-5g) were purchased from Sigma Aldrich (St. Louis, MO, USA). Unless otherwise stated, all the other chemicals and reagents were of analytical grade and used as supplied.

### 4.2. In Vitro Antioxidant Activity

The antioxidant activity of royal jelly was evaluated by diphenyl-1-picrylhydrazyl (DPPH) [43] and ferric ion reducing (FRA) assay [44], as described previously. For the DPPH free radical scavenging assay, 10 μL of varied concentrations of royal jelly (dissolved in water) was mixed with 190 μL of diphenyl-1-picrylhydrazyl solution (DPPH, 24 mg/L in methanol) to obtain the final concentration of 1.5, 3, 6, and 12 mg/mL royal jelly. Simultaneously, a control DPPH solution devoid of royal jelly was prepared. The absorbance of 517 nm was recorded using a Spectrophotometer (UV-2600i-Shimadzu, Kyoto, Japan).

For the FRA assay, 20 μL of varying concentrations of royal jelly were combined separately with 180 μL of FRA reagent. The FRA reagent was prepared by mixing sodium acetate (0.3 M, pH 3.6) with 2,4,6-tripyridyl-S triazin (0.01 M) and ferric chloride (0.02 M) in a 10:1:1 (*v*/*v*). The final concentrations of royal jelly that were tested were 1.5, 3, 6, and 12 mg/mL. Additionally, a control FRA solution was also prepared without the inclusion of royal jelly. The FRA was determined by taking absorbance at 593 nm using a Spectrophotometer (UV-2600i-Shimadzu, Kyoto, Japan).

### 4.3. Zebrafish Husbandry

Zebrafish were maintained at a constant water temperature (28 °C), with a periodic light and dark cycle of 14 h and 10 h, respectively, in the automated water circulation system (Bioengineering Company, Daejeon, Republic of Korea), adhered to the criteria regarding the care and use of laboratory animals [45,46] implemented by the Animal Care Committee and the Use of Raydel Research Institute (approval code RRI-20-003). Normal tetrabit was used to nourish the zebrafish.

### 4.4. Zebrafish Embryo Collection and Evaluation of Embryo-Protective Effect of Royal Jelly

Zebrafish embryos were produced following a previously described method [47]. In brief, male, and female zebrafish were segregated in the water tank using a perforated physical divider. After 16 h segregation, mating was allowed via removal of the physical barrier. The produced embryos were collected, washed, and randomly divided into 6 groups (*n* = 100/group) in each petri dish (100 mm diameter). The embryos in group I were suspended in the 5 mL sea salt solution (3 g sea salt suspended in 100 mL of 1 μg/mL methylene blue) (control). In contrast, the embryos in group II were suspended in a 5 mL sea salt solution containing 0.04% hydrogen peroxide (H_2_O_2_). Embryos in groups III, IV, V, and VI were suspended in 5 mL of 0.04% H_2_O_2_ containing sea salt solution supplemented with 0.25, 0.5, 1, and 2 mg/mL royal jelly, respectively. Embryos in the distinct groups were visualized under a microscope (Motic SMZ 168; Hong Kong) to evaluate embryo mortality.

### 4.5. Microinjection of Zebrafish Embryos

As outlined in Section 4.4, the collected embryos were randomly distributed into 5 distinct groups (*n* = 100 in each group). Embryos in group I received a microinjection of 8 nL PBS (vehicle). Embryos in group II were injected with 500 ng carboxymethyl lysine (CML) suspended in 8 nL PBS. Embryos in groups III, IV, and IV were co-injected with 8 nL of 500 ng CML containing 15, 30, and 60 ng of royal jelly (final), respectively. Microinjection was performed under a microscope using microcapillary pipettes attached with a pneumatic pump (PV830; World Precision Instruments, Sarasota, FL, USA) and a magnetic manipulator (MM33; Kantec, Bensenville, Chicago, IL, USA). The microinjection targeted nearly the same position on the embryo yolk to mitigate bias. The microinjected embryos across all the groups were periodically (0–48 h) visualized under the microscope to assess embryo mortality and developmental defects.

### 4.6. Fluorescent Staining for ROS and Apoptosis

ROS levels in the embryos were determined by dihydroethidium (DHE) fluorescent staining, as in the previously described method [47]. In brief, 10 embryos (5 h post-treatment) were washed with water and immersed in 500 μL of 30 μM DHE solution. After 30 min incubation, embryos were visualized under a fluorescent microscope (Nikon Eclipse TE2000, Tokyo, Japan) at the excitation and emission wavelengths of 585 nm and 615 nm, respectively. The extent of apoptosis was measured via acridine orange (AO) staining, as in a previously described method [47]. In brief, 10 embryos were suspended in 500 μL of 5 μg/mL AO solution. After 1 h incubation, the stained embryos were observed under a fluorescent microscope at 505 nm and 535 nm excitation and emission wavelengths, respectively. The fluorescent intensity of DHE- and AO-stained areas corresponding to ROS and apoptosis was quantified using Image J software (version 1.53r, http://rsb.info.nih.gov/ij/ accessed on 30 January 2023).

### 4.7. Zebrafish Fed with Royal Jelly

Zebrafish (15 weeks aged) were randomly divided into 3 distinct groups (*n* = 40 in each group). The group I zebrafish were fed with normal tetrabit (considered as control). In contrast, zebrafish in groups II and III were fed normal tetrabit supplemented with 5% and 10% royal jelly, respectively. The zebrafish in all groups were nurtured in the same environmental conditions for up to 72 weeks. The survivability and body weight of the zebrafish in all groups were observed periodically (0, 24, 48, and 72 weeks). The body weight was evaluated after anaesthetizing zebrafish using 2-phenoxyethanol (final 0.1%).

### 4.8. Effect of Royal Jelly Supplementation on Egg-Laying and Embryo Survivability of Zebrafish

After 72 weeks, the egg-laying capacity and survivability of the embryos produced from the control, 5% and 10% royal-jelly-supplemented groups were determined. The zebrafish mating in separate groups was performed using a method described in Section 2.4. All the eggs laid in the three groups were suspended in sea salt solution for embryo development. The embryo development and survivability 10 days post-fertilization (dpf) were quantified by visualizing the embryos under the microscope (Motic SMZ 168; Hong Kong). 

### 4.9. Histological Analysis

At the end of the 72 weeks’ supplementation, zebrafish (*n* = 10/group) were sacrificed using hypothermic shock [47] and immediately dissected to recover the liver, testis, and ovary. The dissected tissues were preserved in a 10% formalin solution. For the histological analysis, the tissue was dehydrated using ethanol and embedded in paraffin wax following tissue sectioning (7 μm thick) using microtome (Leica, CM1510s, Heidelberg, Germany). The tissue sections from the liver, ovary, and testis were processed for hematoxylin and eosin staining (H&E) [48,49] to evaluate the morphological changes.

### 4.10. Fluorescent Imaging for Reactive Oxygen Species (ROS) and Apoptosis

The reactive oxygen species (ROS) levels in the tissue section were evaluated by dihydroethidium (DHE) fluorescent staining, as in the previously described method [50]. In brief, the tissue section (7 μm thick) was covered with 250 μL of DHE (30 μM). After 30 min incubation in the dark, the stained section was washed with water and visualized under fluorescent microscopy (Nikon Eclipse TE2000, Tokyo, Japan) at the excitation and emission wavelengths of 588 nm and 605 nm, respectively.

The acridine orange (AO) fluorescent staining was utilized to determine the extent of apoptosis, as in a previously described method [51,52]. In brief, the tissue section (7 μm thick) was covered with 250 μL of AO solution (5 μg/mL) following 30 min incubation in the dark; the stained section was washed with water and visualized at the excitation and emission wavelengths of 502 nm and 525 nm, respectively, using a fluorescent microscope (Nikon Eclipse TE2000, Tokyo, Japan).

### 4.11. Blood Lipid Profile and Hepatic Function Biomarker Analysis

Two microliters of the blood were withdrawn from the zebrafish’s heart and collected in the tubes containing 3 μL of ethylenediaminetetraacetic acid (EDTA, final concentration 1 mM). The serum was collected and processed for the quantification of total cholesterol (TC), triglycerides (TG), and high-density lipoprotein (HDL-C) using a commercial lipid profile diagnostic kit (cholesterol, T-CHO, and TGs, Cleantech TS-S; Walko Pure Chemical, Osaka, Japan). For TC and TG quantification, 5 μL serum was mixed individually with 200 μL of TC- and TG-specific reaction mixture (provided with the kit); after 10 min incubation at 37 °C, TC and TG levels were quantified by measuring absorbance at 490 nm. The HDL-C level was determined by mixing an equal volume of serum and separation solution (provided with the kit). After centrifugation (3000 rpm for 10 min), 20 μL supernatant was mixed with 200 μL of the reaction mixture (provided with the kit), and the HDL-C was quantified by taking absorbance at 490 nm. 

The commercial assay kit (Asan Pharmaceutical, Hwasung, Republic of Korea) was utilized to quantify the serum level of aspartate transaminase (AST) and alanine transaminase (ALT), following the methodology advised by the manufacturers. 

### 4.12. Statistical Analysis

The experiments were conducted in triplicate, and the outcomes are expressed as the mean ± standard deviation. Statistical significance among the groups was assessed through a one-way analysis of variance (ANOVA), following Tukey’s post hoc analysis, employing the Statistical Package for the Social Sciences (SPSS, Chicago, IL, USA, version 29).

## 5. Conclusions

**Figure 11:**
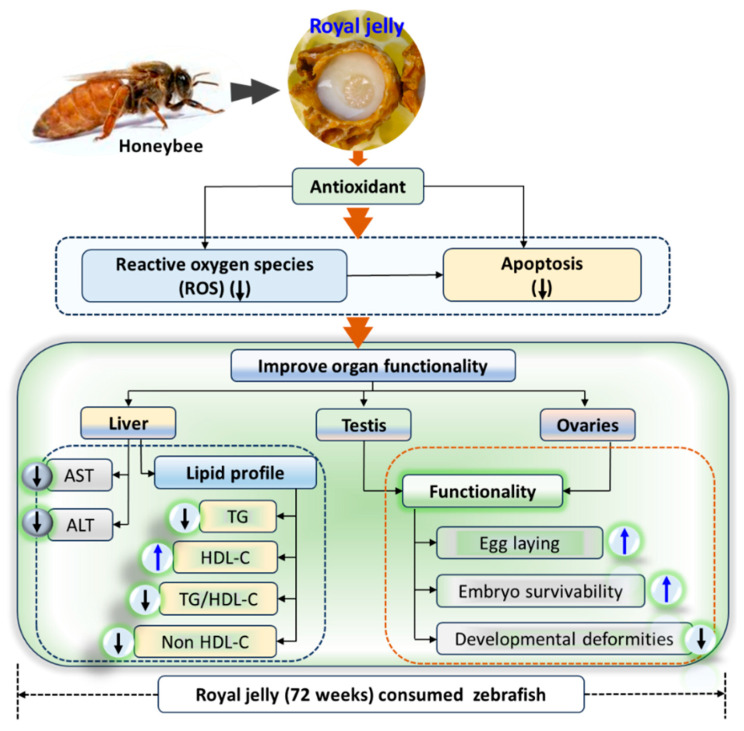
Summary of the various events in zebrafish after 72 weeks of royal jelly consumption. AST, aspartate aminotransferase; ALT, alanine aminotransferase; HDL-C, high-density lipoprotein-cholesterol; ROS, reactive oxygen species; TC, total cholesterol; TG, triglyceride.

Royal jelly was found to have a substantial antioxidant activity that rescues zebrafish embryos from oxidative injury. The dietary consumption of royal jelly for a prolonged period (72 weeks) was found to be safe, without any visible sign of toxicity to the hepatic, ovary, and testis. Furthermore, royal jelly displayed a positive impact on hepatic and sexual health and improved blood lipid profile via elevating HDL-C levels. The most noteworthy effect of royal jelly supplementation was observed on female zebrafish’s weight; however, a detailed mechanism of the body weight gain and change in adipose tissue during the consumption needs to be performed in a future study. The antioxidant nature of royal jelly was regarded as a critical mediator in improving the health-beneficial effects (Figure 11). The findings support the use of royal jelly as a safe dietary supplement, with weight enhancement occurring during long-term supplementation, particularly in the female zebrafish.

## Data Availability

Data is contained within the article and Supplementary Material.

## Supplementary Materials

The following supporting information can be downloaded at: https://www.mdpi.com/article/10.3390/ph17030324/s1, Table S1: Ingredients composition analysis of the royal jelly (Raydel^®^).
