# Supplementary material for: Long-Term Supplementation of Royal Jelly (Raydel®) Improves Zebrafish Growth, Embryo Production and Survivability, Blood Lipid Profile and Functionality of Vital Organs: A 72-Weeks’ Consumption Study
Source: Pharmaceuticals (Basel). 2024 Mar 1;17(3):324. doi: 10.3390/ph17030324 (PMC10976271; doi:10.3390/ph17030324)
Supplement: Supplementary file 1 [file pharmaceuticals-17-00324-s001.zip › pharmaceuticals-2859828-supplementary.pdf]

Supplementary material

Table S1. Ingredients composition analysis of the royal jelly (Raydel®)

Product Name/Description	Fresh Royal jelly
**Country**	Shanghai, China
**Manufacturer**	Wuxi Long life Healthcare food
**Ingredient**	100% fresh royal jelly
**Excipient**	None
**Ingredients of Royal jelly**	**Determined Amount (%)**
Total amount on the label	100
Moisture	65.94
Protein	14.15
Total carbohydrate	11.58
Fat	5.23
10-hydroxydecanoic acid (10-HAD)	1.64
Minerals	0.77
Undetermined	1
Sodium (mg/100g)	96
Energy (Kcal/100g)	162
Coliform Group bacteria (CFU/g)	<10
Total bacteria count (CFU/g)	<10
Chloramphenicol	Not detected
Nitrofurans	Not detected
